# Supplementary material for: Comparative optimization of polysaccharide-based nanoformulations for cardiac RNAi therapy
Source: Nat Commun. 2024 Jun 26;15:5398. doi: 10.1038/s41467-024-49804-x (PMC11208445; doi:10.1038/s41467-024-49804-x)
Supplement: Supplementary file 1 — Supporting information [file 41467_2024_49804_MOESM1_ESM.pdf]

Supplementary Materials for

Comparative optimization of polysaccharide-based nanoformulations for cardiac RNAi therapy

Han Gao, Sen Li, Zhengyi Lan, Da Pan, Gonna Somu Naidu, Dan Peer,
Chenyi Ye, Hangrong Chen, Ming Ma*, Zehua Liu*, Hélder A. Santos*

*Corresponding author. Email: mma@mail.sic.ac.cn; zehua.liu@helsinki.fi; h.a.santos@umcg.nl

This PDF file includes:

Figs. S1 to S16
Tables S1 to S8

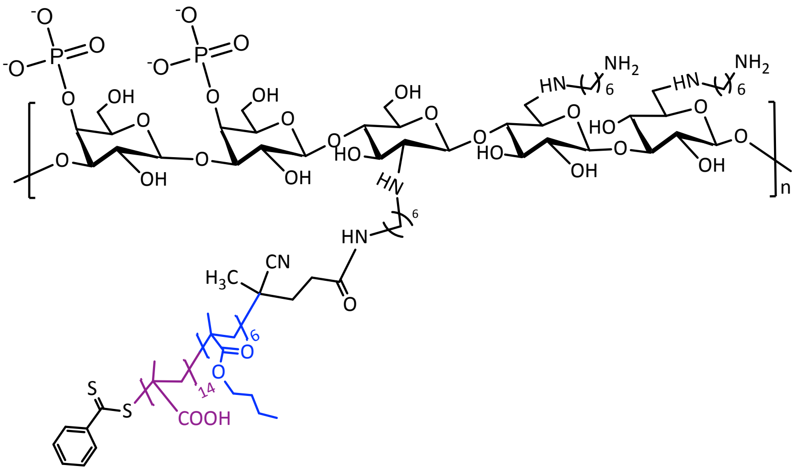

Fig. S1. Structure of endosomolytic phosphorylated β-glucan derivative, EEPG.

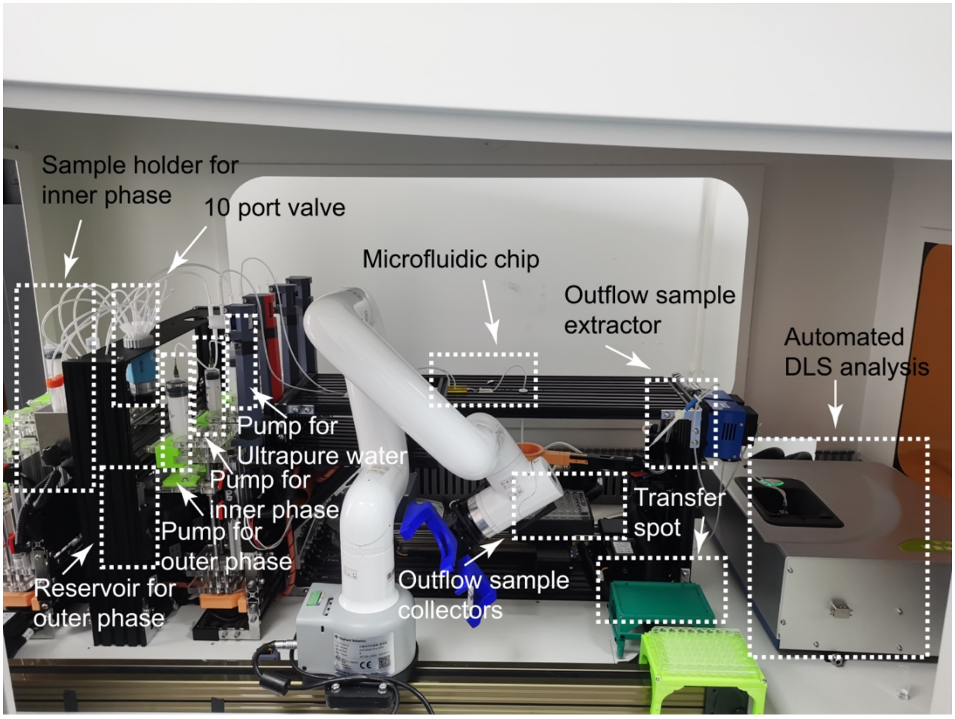

Fig. S2. The representative schematic of the pre-mixed automatic microfluidics system.

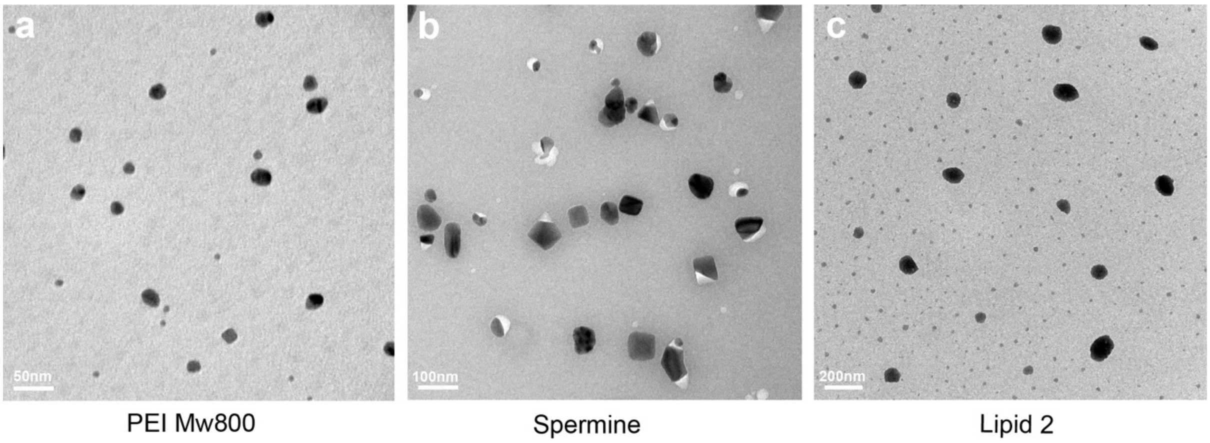

Fig. S3. Representative TEM images from each cationic species-composed nanosystems. (a) PEI (Mw = 800)-EEPG NPs. (b) Spermine-EEPG NPs. (c) Lipid 2-EEPG NPs.

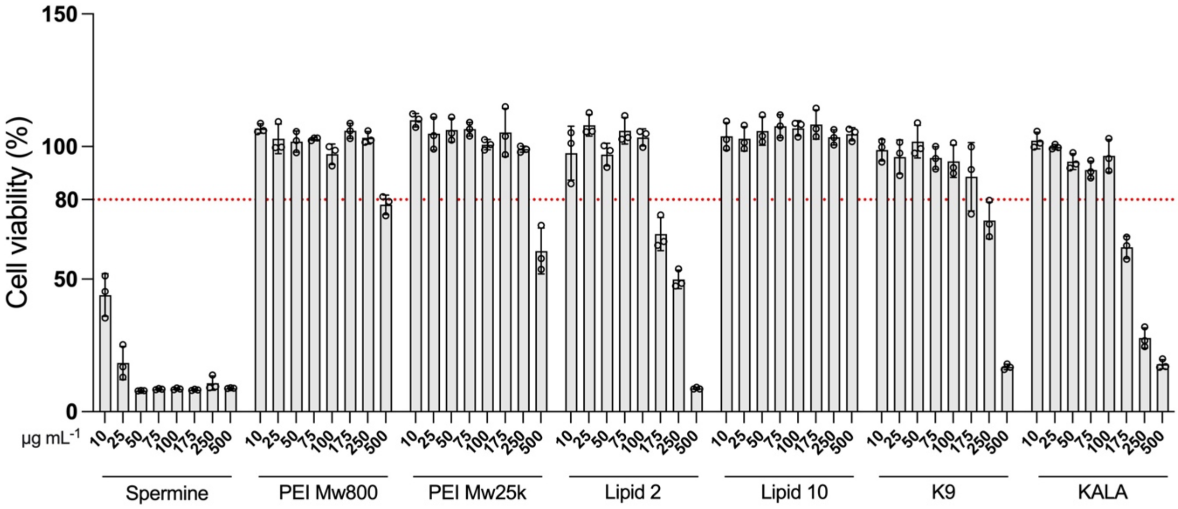

Fig. S4. Cell viability on RAW 264.7 cells after treatment with representative nanosystems for 48 h (n=3 replicates). Data are presented as mean ± SD. Source Data are provided in the Source Data File.

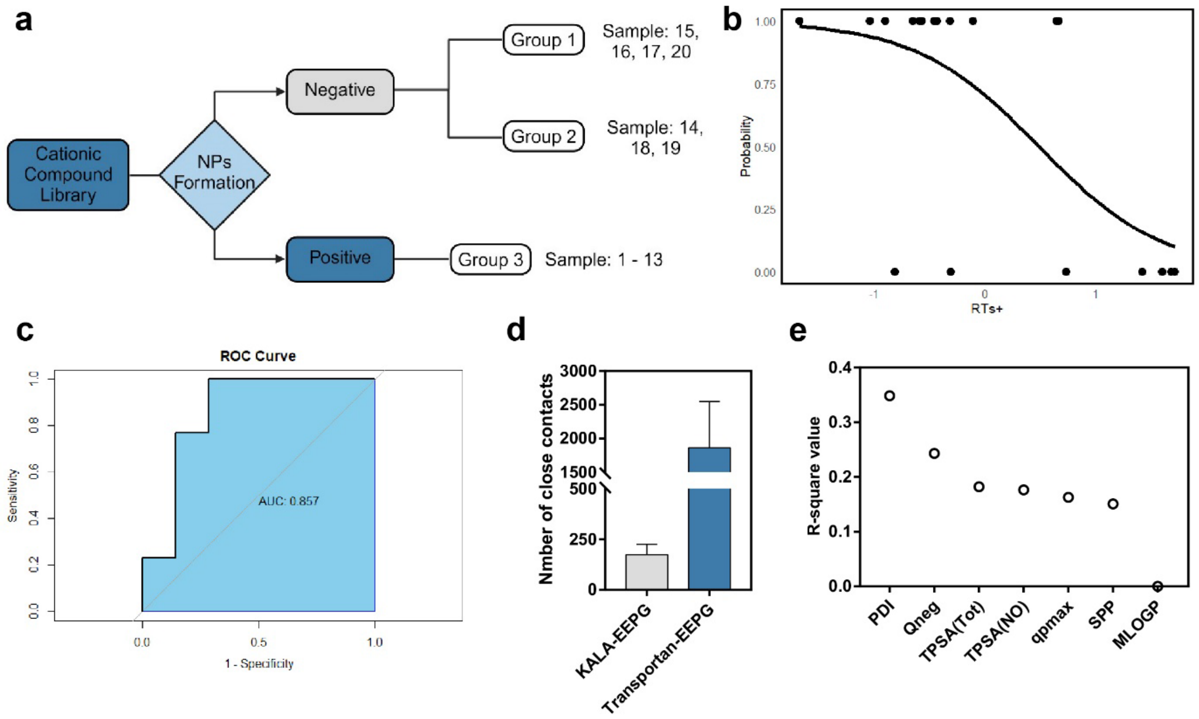

Fig. S5. Computational assisted step-wise interpretation of formulation screening process. (a) Categories of different samples in the screening process for determining the NPs size and formation. (b) RTs+ (R maximal index weighted by intrinsic-state) of MolDes of each cationic compound. (c) The corresponding ROC curve of MolDes with the lowest p-value. (d) Molecular dynamic simulation was performed to compare the number of contacts in KALA-EEPG NPs and Transportan-EEPG NPs. (e) R-square values from MolDes describing hydrophobicity, polar surface area and positive charges.

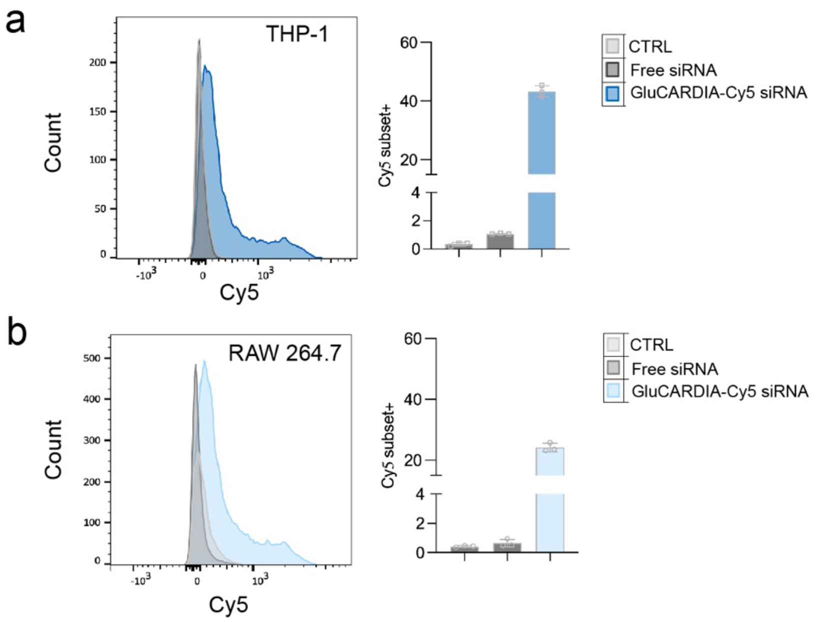

Fig. S6. *In vitro* cellular uptake of GluCARDIA-Cy5 siRNA. (a) Quantitative analysis of Cy5 siRNA uptake in THP-1 cells (n=3, replicates). (b) Quantitative analysis of Cy5 siRNA uptake in RAW 264.7 cells (n=3, replicates). Data are presented as mean ± SD. Source Data are provided in the Source Data File.

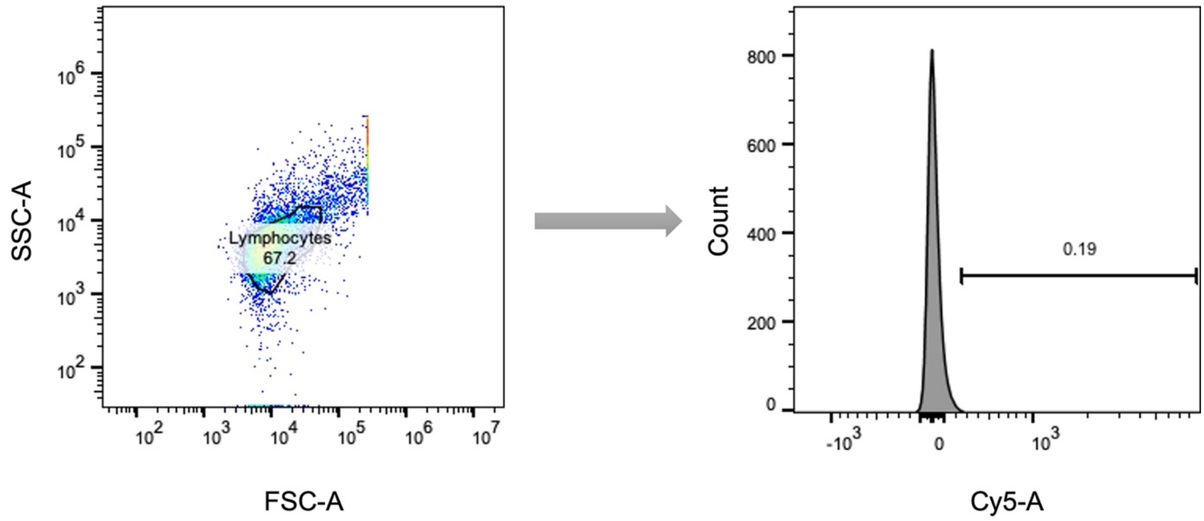

Fig. S7. Flow cytometry gating strategy. Cells were gated to identify Cy5-siRNA signal inside cells.

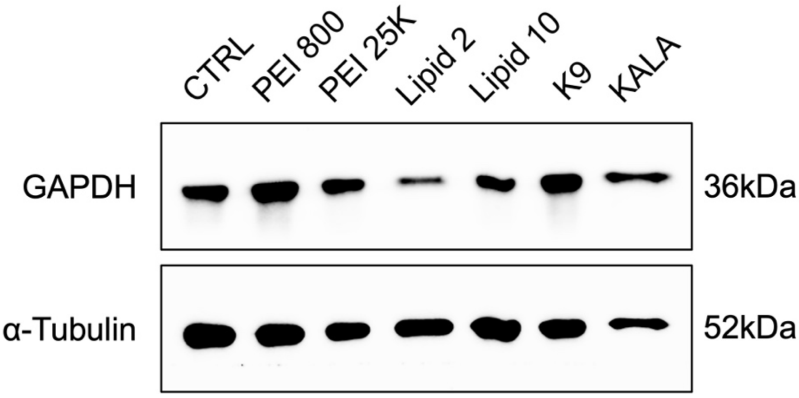

Fig. S8. GAPDH knockdown on RAW 264.7 cells at 48 h post-treatment with representative nanosystems, siGAPDH final concentration at 50 nM (n=3 per group). Source Data are provided in the Source Data File.

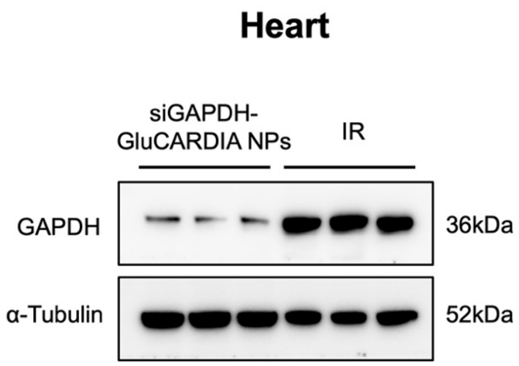

Fig. S9. Western blotting analysis was performed to evaluate the gene silencing efficacy of GluCARDIA-siGAPDH NPs in heart and liver (n=3 mice). Source Data are provided in the Source Data File.

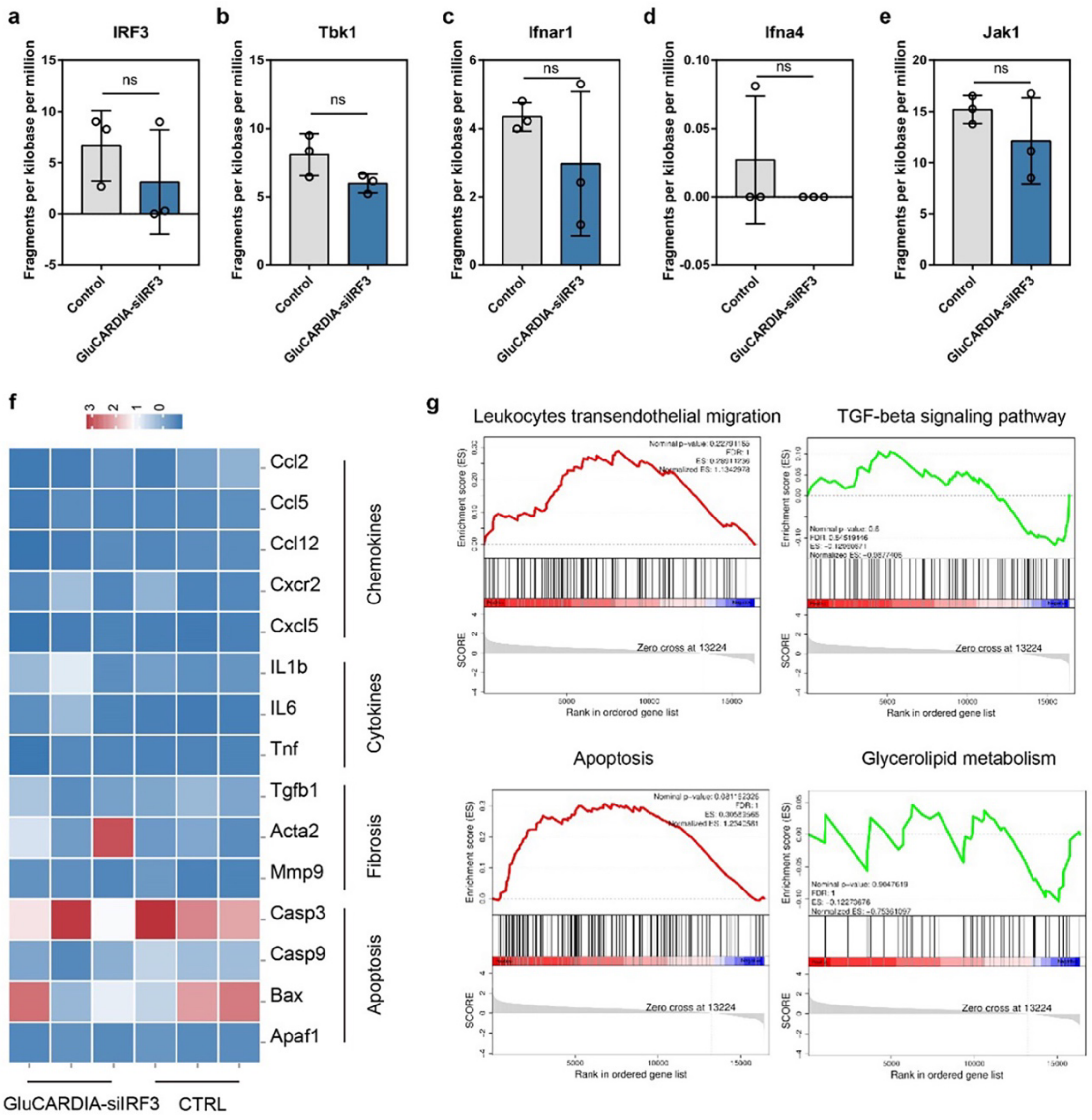

Fig. S10. (a-e) Expression level of Irf3 and Irf3 related genes including Tbk1, Ifnar1, Ifna4 and Jak between IR and GluCARDIA-siIRF3 group (n=3, mice); (f) Heat map of chemokines, cytokines, fibrosis progression and apoptosis-related gene expressions. (g) GSEA for indicated Gene Ontology (GO) or Kyoto Encyclopedia of Genes and Genomes (KEGG) defined gene clusters.

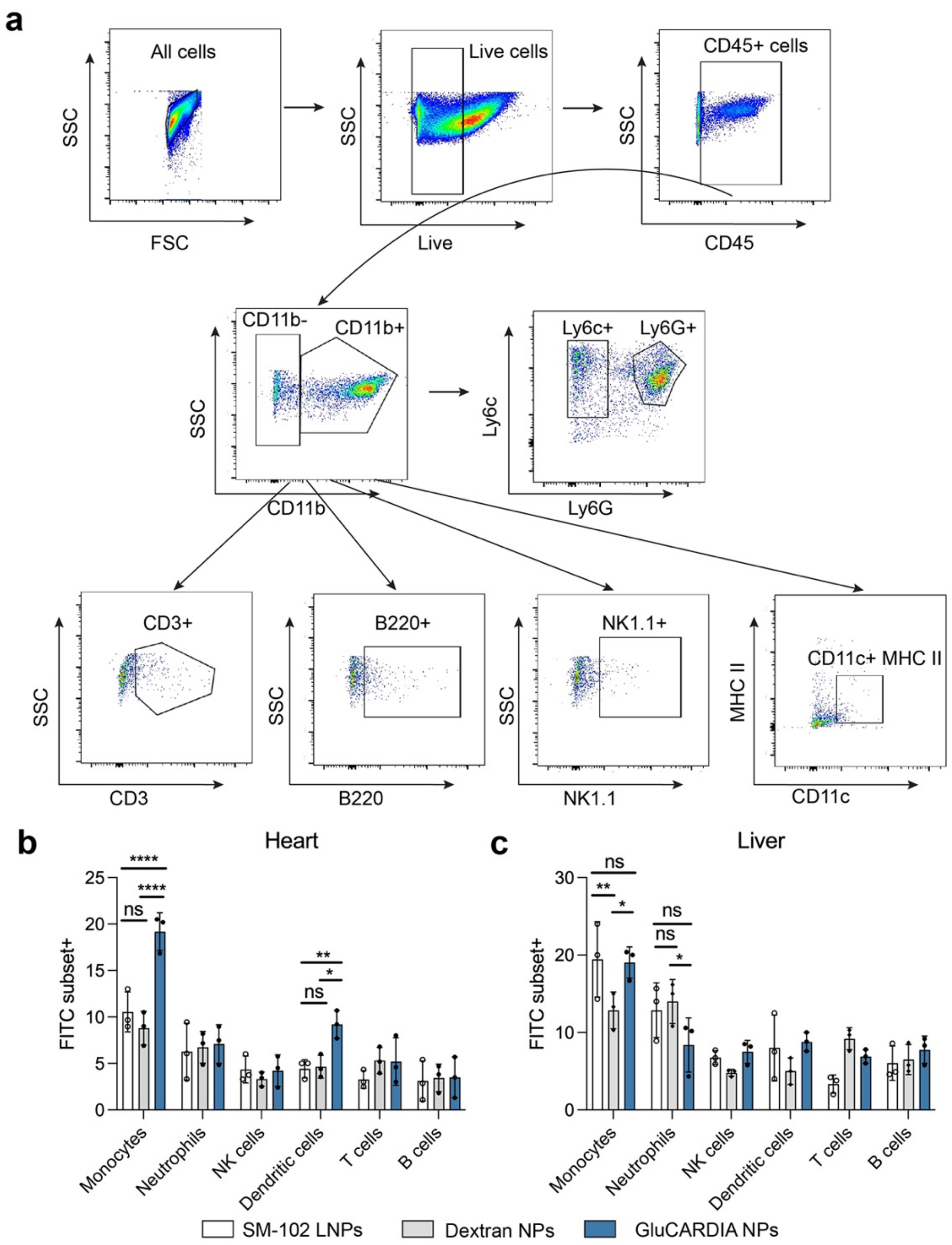

Fig. S11. Flow cytometry quantification of subcellular uptake of different NPs in immune cell subsets from heart and liver. (a) Representative illustration for gating strategy. All cells were extracted from the heart and liver, and further labeled with antibodies as described in Methods. Monocytes was identified as CD45+CD11b+Ly6G-Ly6clow to high, neutrophils were identified as CD45+CD11b+Ly6G+, T cells were identified as CD45+CD11b-CD3+, B cells were identified as CD45+CD11b-B220+, NK cells were identified as CD45+CD11b-NK1.1+, DCs were identified as CD45+CD11b-CD11c+MHC II+. Quantitative analysis of immune cell uptake of SM-102 LNPs/Dextran NPs/GluCARDIA NPs was performed on (b) heart and (c) liver (n=3 mice). Data are presented as mean ± SD. ns: no significance; *, $p < 0.05$, **, $p < 0.01$, ****, $p < 0.0001$. Source Data are provided in the Source Data File.

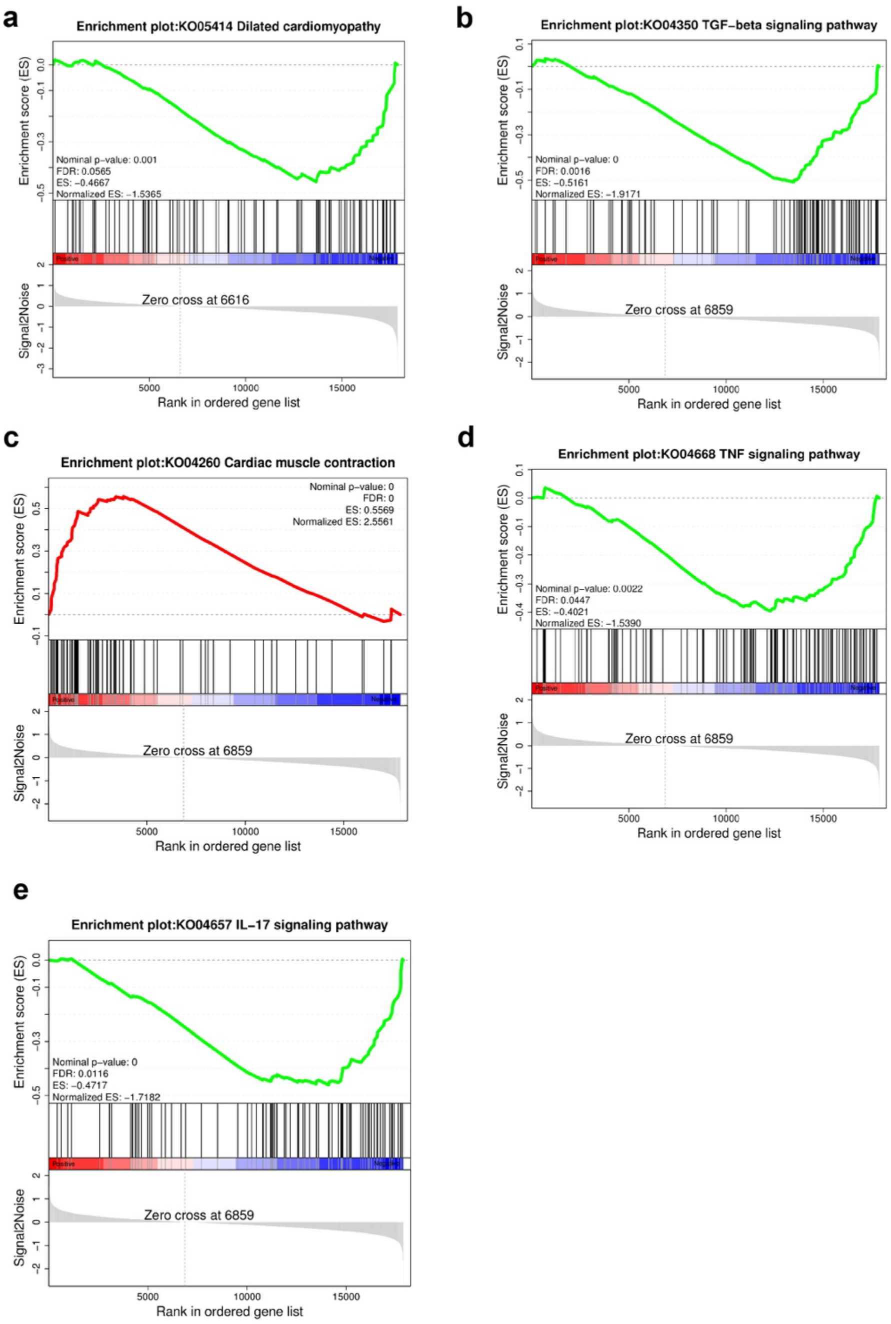

Fig. S12. Gene Set Enrichment Analysis (GSEA) was performed based on the RNA-seq datasets. Positive and negative enrichment score indicate higher and lower expression respectively. (a) IR *vs.* GluCARDIA-siIRF3, negative association between GluCARDIA-siIRF3 treatment and dilated cardiomyopathy was observed. (b) TGF-β pathway analysis, (c) cardiac muscle contraction analysis, (d) TNF pathway analysis, and (e) IL-17 pathway analysis showed GluCARDIA-siIRF3 may inhibited fibrosis progress, promoted cardiac contraction and facilitated the inflammation resolution, comparing to GluCARDIA-siNC group.

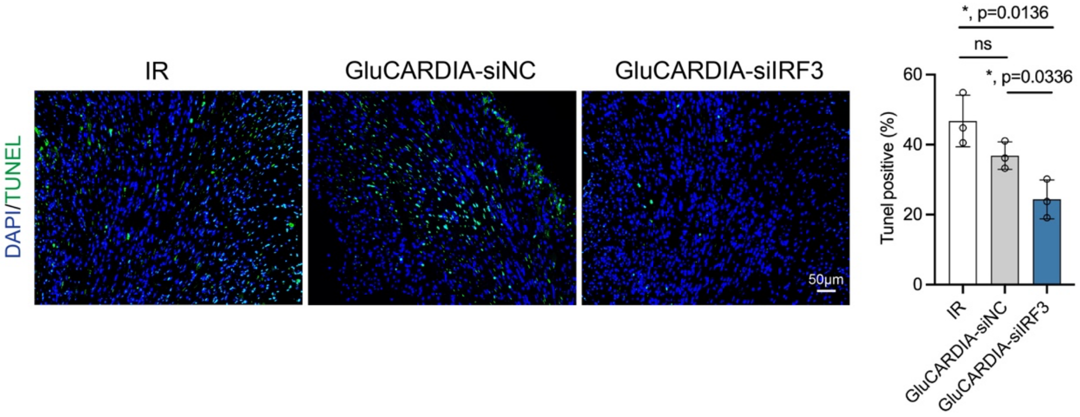

Fig. S13. Representative TUNEL staining of cardiac myocytes in heart sections from IR and GluCARDIA-siIRF3 NPs (n=3 mice). Scale bar: 50 μm. Source Data are provided in the Source Data File.

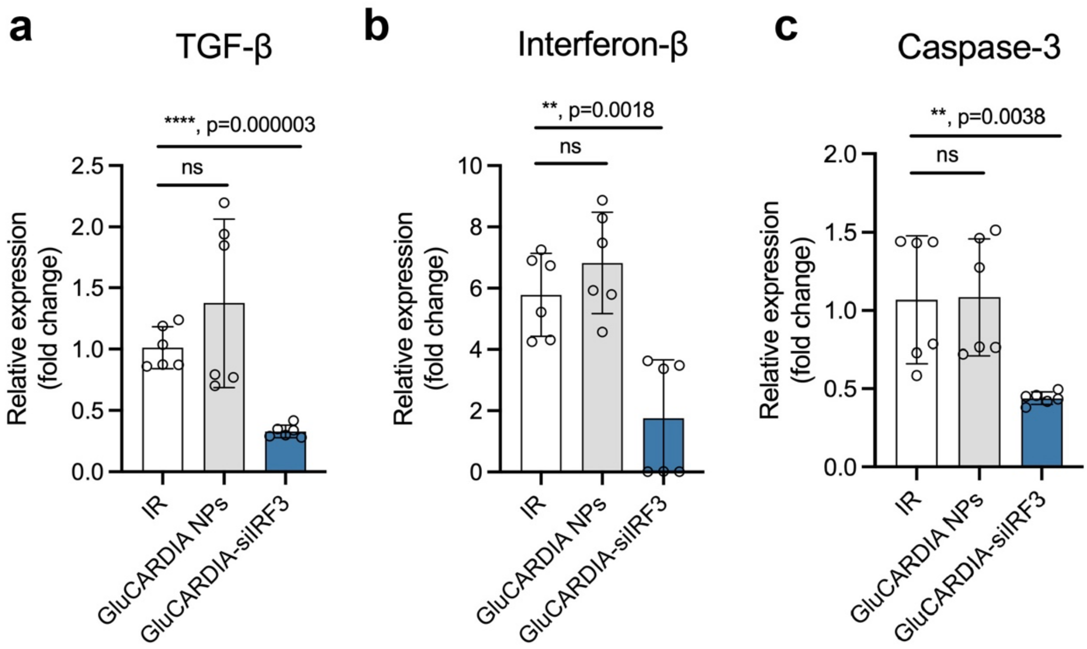

Fig. S14. RT-qPCR was used to detect the expression levels of (a) TGF-β, (b) Interferon-β and (c) Caspase-3 in mice from each group. Data are presented as mean ± SD. ns: not significant; **p*<0.05; ***p*<0.01; ****p*<0.001; *****p*<0.0001. Source Data are provided in the Source Data File.

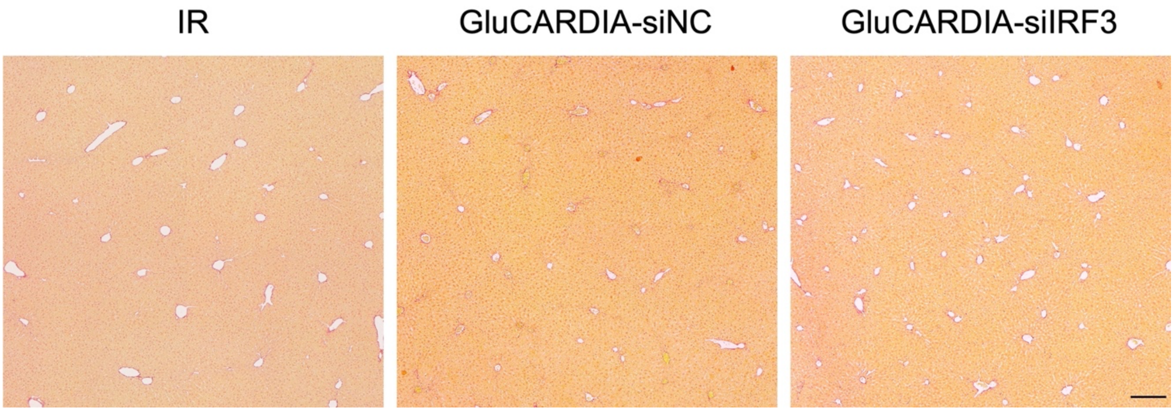

Fig. S15. Representative Sirius Red Staining images of liver sections day 28 post-i.v. injection (n=3 per group). Scale bar: 200 μm.

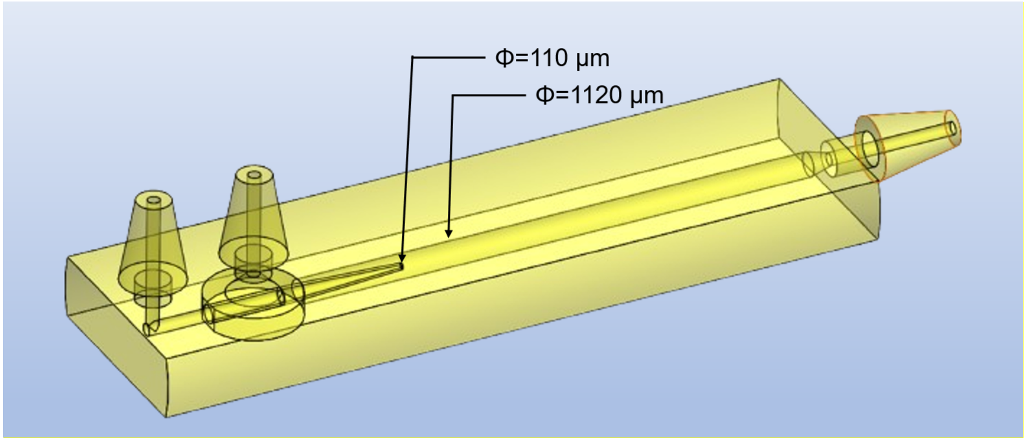

Fig. S16. Schematic diagram illustrating the dimensions of the co-flow focusing microfluidic chip. The inner tube outlet possesses an inner diameter of 110 μm, whereas the outer tube has an inner diameter of 1120 μm.

Table S1. Synthesis conditions for EEPG-focused screen.

Materials ID	Cationic materials' stock solutions	EEPG stock solution	Ratios (Cationic materials: EEPG) (range)	Flow rates (range)
#C1 1	0.5 mg mL^{-1} in Tris-HCL	Tris-HCL 0.5 mg mL^{-1}	0.3/1	Inner phase: 20 mL h^{-1} Outer phase: 20 mL h^{-1}
	1mg mL^{-1} in Tris-HCL		0.4/1	
	2mg mL^{-1} in Tris-HCL		0.6/1	
	3mg mL^{-1} in Tris-HCL		0.8/1	
	4mg mL^{-1} in Tris-HCL		1/1	
	5mg mL^{-1} in Tris-HCL		2/1	
	6mg mL^{-1} in Tris-HCL		3/1	
#C1 2	0.5mg mL^{-1} in Tris-HCL	Tris-HCL 0.5 mg mL^{-1}	0.3/1	
	1mg mL^{-1} in Tris-HCL		0.4/1	
	2mg mL^{-1} in Tris-HCL		0.6/1	
	3mg mL^{-1} in Tris-HCL		0.8/1	
	4mg mL^{-1} in Tris-HCL		1/1	
	5mg mL^{-1} in Tris-HCL		2/1	
	6mg mL^{-1} in Tris-HCL		3/1	
#C1 3	0.25mg mL^{-1} in 0.1M HAC	1 M NaOH 1 mg mL^{-1}	0.3/1	
	0.3mg mL^{-1} in 0.1M HAC		0.4/1	
	0.6mg mL^{-1} in 0.1M HAC		0.6/1	
	0.5mg mL^{-1} in 0.1M HAC		0.8/1	
	0.75mg mL^{-1} in 0.1M HAC		1/1	
	1mg mL^{-1} in 0.1M HAC		2/1	
	2mg mL^{-1} in 0.1M HAC		3/1	
#C1 4	0.25mg mL^{-1} in 0.1M HAC	1 M NaOH 1 mg mL^{-1}	0.3/1	
	0.3mg mL^{-1} in 0.1M HAC		0.4/1	
	0.6mg mL^{-1} in 0.1M HAC		0.6/1	
	0.5mg mL^{-1} in 0.1M HAC		0.8/1	
	0.75mg mL^{-1} in 0.1M HAC		1/1	
	1mg mL^{-1} in 0.1M HAC		2/1	
	2mg mL^{-1} in 0.1M HAC		3/1	
#C1 5	0.25mg mL^{-1} in 0.1M HAC	1 M NaOH 1 mg mL^{-1}	0.3/1	
	0.3mg mL^{-1} in 0.1M HAC		0.4/1	
	0.6mg mL^{-1} in 0.1M HAC		0.6/1	
	0.5mg mL^{-1} in 0.1M HAC		0.8/1	
	0.75mg mL^{-1} in 0.1M HAC		1/1	
	1mg mL^{-1} in 0.1M HAC		2/1	
	2mg mL^{-1} in 0.1M HAC		3/1	
#C1 6	0.2mg mL^{-1} in Tris-HCL	Tris-HCL 2 mg mL^{-1}	0.3/1	
	0.25mg mL^{-1} in Tris-HCL		0.4/1	
	0.4mg mL^{-1} in Tris-HCL		0.6/1	
	0.5mg mL^{-1} in Tris-HCL		0.8/1	
	0.67mg mL^{-1} in Tris-HCL		1/1	
	1mg mL^{-1} in Tris-HCL		2/1	
	2mg mL^{-1} in Tris-HCL		3/1	

#C1 7	0.2mg mL^{-1} in Tris-HCL	Tris-HCL 2 mg mL^{-1}	0.3/1	
	0.25mg mL^{-1} in Tris-HCL		0.4/1	
	0.4mg mL^{-1} in Tris-HCL		0.6/1	
	0.5mg mL^{-1} in Tris-HCL		0.8/1	
	0.67mg mL^{-1} in Tris-HCL		1/1	
	1mg mL^{-1} in Tris-HCL		2/1	
	2mg mL^{-1} in Tris-HCL		3/1	
#C2 8	3.2 mg mL^{-1} in 0.1M HAC	1 M NaOH 0.4 mg mL^{-1}	8:1	
	1.6 mg mL^{-1} in 0.1M HAC		4:1	
	1.2 mg mL^{-1} in 0.1M HAC		3:1	
	0.8 mg mL^{-1} in 0.1M HAC		2:1	
	0.4 mg mL^{-1} in 0.1M HAC		1:1	
	0.2 mg mL^{-1} in 0.1M HAC		1:2	
	0.1 mg mL^{-1} in 0.1M HAC		1:4	
#C2 9	3.2 mg mL^{-1} in 0.1M HAC	1 M NaOH 0.4 mg mL^{-1}	8:1	
	1.6 mg mL^{-1} in 0.1M HAC		4:1	
	1.2 mg mL^{-1} in 0.1M HAC		3:1	
	0.8 mg mL^{-1} in 0.1M HAC		2:1	
	0.4 mg mL^{-1} in 0.1M HAC		1:1	
	0.2 mg mL^{-1} in 0.1M HAC		1:2	
	0.1 mg mL^{-1} in 0.1M HAC		1:4	
#C3 10	0.2mg mL^{-1} in Tris-HCL	Tris-HCL 0.4 mg mL^{-1}	1:2	
	0.4mg mL^{-1} in Tris-HCL		1:1	
	0.8mg mL^{-1} in Tris-HCL		2:1	
	1.6mg mL^{-1} in Tris-HCL		4:1	
	3.2mg mL^{-1} in Tris-HCL		8:1	
	4mg mL^{-1} in Tris-HCL		10:1	
	6mg mL^{-1} in Tris-HCL		15:1	
#C3 11	0.2mg mL^{-1} in Tris-HCL	Tris-HCL 0.4 mg mL^{-1}	1:2	
	0.4mg mL^{-1} in Tris-HCL		1:1	
	0.8mg mL^{-1} in Tris-HCL		2:1	
	1.6mg mL^{-1} in Tris-HCL		4:1	
	3.2mg mL^{-1} in Tris-HCL		8:1	
	4mg mL^{-1} in Tris-HCL		10:1	
	6mg mL^{-1} in Tris-HCL		15:1	
#C3 12	0.2mg mL^{-1} in Tris-HCL	Tris-HCL 0.4 mg mL^{-1}	1:2	
	0.4mg mL^{-1} in Tris-HCL		1:1	
	0.8mg mL^{-1} in Tris-HCL		2:1	
	1.6mg mL^{-1} in Tris-HCL		4:1	
	3.2mg mL^{-1} in Tris-HCL		8:1	
	4mg mL^{-1} in Tris-HCL		10:1	
	6mg mL^{-1} in Tris-HCL		15:1	
#C3 13	0.2mg mL^{-1} in Tris-HCL	Tris-HCL 0.4 mg mL^{-1}	1:2	
	0.4mg mL^{-1} in Tris-HCL		1:1	
	0.8mg mL^{-1} in Tris-HCL		2:1	
	1.6mg mL^{-1} in Tris-HCL		4:1	
	3.2mg mL^{-1} in Tris-HCL		8:1	
	4mg mL^{-1} in Tris-HCL		10:1	

	6mg mL^{-1} in Tris-HCL		15:1	
#C4 14	0.1mg mL^{-1} in ETOH:0.1M HAC (v/v 1:3)	1 M NaOH 0.4 mg mL^{-1}	1:4	
	0.1mg mL^{-1} in ETOH:0.1M HAC (v/v 1:3)		1:3	
	0.1mg mL^{-1} in ETOH:0.1M HAC (v/v 1:3)		1:2	
	0.1mg mL^{-1} in ETOH:0.1M HAC (v/v 1:3)		1:1	
	0.1mg mL^{-1} in ETOH:0.1M HAC (v/v 1:3)		2:1	
	0.15mg mL^{-1} in ETOH:0.1M HAC (v/v 1:3)		4:1	
	0.3mg mL^{-1} in ETOH:0.1M HAC (v/v 1:3)		8:1	
#C4 15	0.1mg mL^{-1} in ETOH:0.1M HAC (v/v 1:3)	1 M NaOH 0.4 mg mL^{-1}	1:4	
	0.1mg mL^{-1} in ETOH:0.1M HAC (v/v 1:3)		1:3	
	0.1mg mL^{-1} in ETOH:0.1M HAC (v/v 1:3)		1:2	
	0.1mg mL^{-1} in ETOH:0.1M HAC (v/v 1:3)		1:1	
	0.1mg mL^{-1} in ETOH:0.1M HAC (v/v 1:3)		2:1	
	0.15mg mL^{-1} in ETOH:0.1M HAC (v/v 1:3)		4:1	
	0.3mg mL^{-1} in ETOH:0.1M HAC (v/v 1:3)		8:1	
#C4 16	0.1mg mL^{-1} in ETOH:0.1M HAC (v/v 1:3)	1 M NaOH 0.4 mg mL^{-1}	1:4	
	0.1mg mL^{-1} in ETOH:0.1M HAC (v/v 1:3)		1:3	
	0.1mg mL^{-1} in ETOH:0.1M HAC (v/v 1:3)		1:2	
	0.1mg mL^{-1} in ETOH:0.1M HAC (v/v 1:3)		1:1	
	0.1mg mL^{-1} in ETOH:0.1M HAC (v/v 1:3)		2:1	
	0.15mg mL^{-1} in ETOH:0.1M HAC (v/v 1:3)		4:1	
	0.3mg mL^{-1} in ETOH:0.1M HAC (v/v 1:3)		8:1	
#C4 17	0.1mg mL^{-1} in ETOH:0.1M HAC (v/v 1:3)	1 M NaOH 0.4 mg mL^{-1}	1:4	
	0.1mg mL^{-1} in ETOH:0.1M HAC (v/v 1:3)		1:3	
	0.1mg mL^{-1} in ETOH:0.1M HAC (v/v 1:3)		1:2	

	0.1mg mL^{-1} in ETOH:0.1M HAC (v/v 1:3)		1:1	
	0.1mg mL^{-1} in ETOH:0.1M HAC (v/v 1:3)		2:1	
	0.15mg mL^{-1} in ETOH:0.1M HAC (v/v 1:3)		4:1	
	0.3mg mL^{-1} in ETOH:0.1M HAC (v/v 1:3)		8:1	
#C4 18	0.1mg mL^{-1} in ETOH:0.1M HAC (v/v 1:3)	1 M NaOH 0.4 mg mL^{-1}	1:4	
	0.1mg mL^{-1} in ETOH:0.1M HAC (v/v 1:3)		1:3	
	0.1mg mL^{-1} in ETOH:0.1M HAC (v/v 1:3)		1:2	
	0.1mg mL^{-1} in ETOH:0.1M HAC (v/v 1:3)		1:1	
	0.1mg mL^{-1} in ETOH:0.1M HAC (v/v 1:3)		2:1	
	0.15mg mL^{-1} in ETOH:0.1M HAC (v/v 1:3)		4:1	
	0.3mg mL^{-1} in ETOH:0.1M HAC (v/v 1:3)		8:1	
#C4 19	0.1mg mL^{-1} in ETOH:0.1M HAC (v/v 1:3)	1 M NaOH 0.4 mg mL^{-1}	1:4	
	0.1mg mL^{-1} in ETOH:0.1M HAC (v/v 1:3)		1:3	
	0.1mg mL^{-1} in ETOH:0.1M HAC (v/v 1:3)		1:2	
	0.1mg mL^{-1} in ETOH:0.1M HAC (v/v 1:3)		1:1	
	0.1mg mL^{-1} in ETOH:0.1M HAC (v/v 1:3)		2:1	
	0.15mg mL^{-1} in ETOH:0.1M HAC (v/v 1:3)		4:1	
	0.3mg mL^{-1} in ETOH:0.1M HAC (v/v 1:3)		8:1	
#C4 20	0.1mg mL^{-1} in ETOH:0.1M HAC (v/v 1:3)	1 M NaOH 0.4 mg mL^{-1}	1:4	
	0.1mg mL^{-1} in ETOH:0.1M HAC (v/v 1:3)		1:3	
	0.1mg mL^{-1} in ETOH:0.1M HAC (v/v 1:3)		1:2	
	0.1mg mL^{-1} in ETOH:0.1M HAC (v/v 1:3)		1:1	
	0.1mg mL^{-1} in ETOH:0.1M HAC (v/v 1:3)		2:1	
	0.15mg mL^{-1} in ETOH:0.1M HAC (v/v 1:3)		4:1	

	0.3mg mL^{-1} in ETOH:0.1M HAC (v/v 1:3)		8:1	

*HAC: Sodium acetate buffer; NaOH: sodium hydroxide buffer; Tris-HCL: Tris hydrochloride buffer.

Table S2. Characterization of polymers-assembled formulations.

C1. Polymers							
**N:P ratios**	**Dex-dia**	**Dex-spermine**	**CS Low Mw**	**CS Med Mw**	**CS Mw 15k**	**PEI Mw 800**	**PEI Mw 25k**
0.3/1	—	—	—	—	—	—	129.23±1.68
0.4/1	—	—	—	—	—	128.53±1.31	136.67±2.93
0.6/1	—	—	—	—	—	104.77±2.29	185.07±0.21
0.8/1	—	—	191.86±3.71	371.77±8.80	137.4±1.59	—	726.90±20.78
1/1	—	—	—	—	—	—	—
2/1	—	—	206.93±2.46	161.23±2.14	—	—	353.27±34.08
3/1	—	—	—	—	—	—	—

* — represents non-obvious NPs formation between oppositely charged species.

Table S3. Characterization of small molecular drugs-assembled formulations.

**C2. Small molecular drugs**		
**Weight ratios**	**Spermine**	**Deferoxamine**
8/1	214.07±14.93	—
4/1	—	—
3/1	—	—
2/1	—	—
1/1	—	—
1/2	—	—
1/4	—	—

* — represents non-obvious NPs formation between oppositely charged species.

Table S4. Characterization of cell-penetrating peptides-assembled formulations.

**C3. Cell-penetrating peptides**				
**Weight ratios**	**K9**	**KALA**	**Transportan**	**Penetratin**
1/2	146.30±0.62	117.30±2.69	144.47±3.23	106.80±2.52
1/1	723.95±193.54	132.23±0.61	136.27±1.42	157.30±2.46
2/1	—	—	—	—
4/1	—	—	—	—
8/1	762.07±119.39	113.93±0.06	—	—
10/1	—	—	—	—
15/1	—	—	—	—

* — represents non-obvious NPs formation between oppositely charged species.

Table S5. Characterization of lipids-assembled formulations.

C4. Lipids							
**Weight ratios**	**Lipid 2**	**Lipid 6**	**Lipid 8**	**Lipid 10**	**Lipid 14**	**DOTAP**	**MC3**
1/4	—	248.13± 6.67	—	—	—	237.00± 4.78	148.53± 3.65
1/3	84.77± 2.12	—	—	—	—	—	—
1/2	154.47± 1.02	710.30± 34.46	—	—	—	214.90± 6.51	146.90± 2.69
1/1	195.2± 3.00	—	—	—	—	—	137.03± 1.74
2/1	178.97± 2.15	290.03± 8.35	—	—	—	213.50± 9.56	513.43± 43.63
4/1	200.83± 4.48	—	—	—	—	—	502.87± 32.01
8/1	213.3± 8.06	—	—	—	—	—	—

* — represents non-obvious NPs formation between oppositely charged species.

Table S6. Characterization of Lipid 8/Lipid 10/Lipid 14-assembled formulations.

**C4. Lipids**			
**Weight ratios**	**Lipid 8**	**Lipid 10**	**Lipid 14**
1/35	169.10± 2.80	—	—
1/30	168.57± 2.12	—	—
1/25	184.20± 2.75	166.53± 4.15	—
1/20	214.13± 2.89	—	—
1/15	658.70± 109.24	223.90± 5.65	—
1/10	—	—	—
1/8	—	—	—

* — represents non-obvious NPs formation between oppositely charged species.

Table S7. Dictionary for MolDes.

**Name**	**Category**	**Description**
qnmax	Charge descriptors	Maximum negative charge
B02[O-O]	2D atom pairs	Presence/absence of O – O at topological distance 2
B10[N-N]	2D atom pairs	Presence/absence of N – N at topological distance 10
SPP	Charge descriptors	Submolecular polarity parameter
GATS8i	2D autocorrelations	Geary autocorrelation of lag 8 weighted by ionization potential
qpmax	Charge descriptors	Maximum positive charge
Qpos	Charge descriptors	Total positive charge
pKa	Molecular properties	pKa (strongest base)
MLOGP	Molecular properties	Moriguchi octanol-water partition coeff. (logP)
TDB10P	3D autocorrelations	3D Topological distance based descriptors – lag 10 weighted by polarizability
VE2sign_G	3D matrix-based descriptors	Average coefficient of the last eigenvector from geometrical matrix
E3m	WHIM descriptors	3rd component accessibility directional WHIM index / weighted by mass
VE2_Coulomb	3D matrix-based descriptors	Average coefficient of the last eigenvector (absolute values) from Coulomb matrix
H2p	GETAWAY descriptors	H autocorrelation of lag 2 / weighted by polarizability
PDI	Molecular properties	Packing density index
Qneg	Charge descriptors	Total negative charge
TPSA(Tot)	Molecular properties	Topological polar surface area using N,O,S,P polar contributions
TPSA(NO)	Molecular properties	Topological polar surface area using N,O polar contributions

Table S8. List of antibodies used in this study.

Antibody specificity	Supplier	Cat. No.
GAPDH monoclonal antibody	Proteintech	60004-1-Ig
HRP-conjugated Alpha Tubulin Monoclonal antibody	Proteintech	HRP-66031
IRF3 monoclonal antibody	Proteintech	66670-1-Ig
Phospho-IRF3 (Ser396) antibody	Affinity Biosciences	AF2436
Recombinant mouse Dectin-1	Abcam	ab217331
Ly-6G	R&D Systems	MAB1037
DAPI	Servicebio	G1012
HRP-conjugated Affinipure Goat Anti-Rabbit IgG(H+L)	Proteintech	SA00001-2
HRP-conjugated Affinipure Goat Anti-Mouse IgG(H+L)	Proteintech	SA00001-1
PE/Cyanine7 anti-mouse CD45	Biolegend	103113
Brilliant Violet 650™ anti-mouse/Human CD11b	Biolegend	101259
PerCP/Cyanine5.5 anti-mouse Ly-6G	Biolegend	127615
Brilliant Violet 421™ anti-mouse Ly-6C	Biolegend	128031
PE-Cy™7 Rat Anti-Mouse CD45R/B220	BD Pharmingen	552772
BV786 Mouse Anti-Mouse NK-1.1	BD Pharmingen	740853
PE anti-mouse CD3ε Antibody	Biolegend	100307
APC anti-mouse CD11c	Biolegend	117309
Zombie NIR	Biolegend	423105
