# Supplementary material for: Comparative optimization of polysaccharide-based nanoformulations for cardiac RNAi therapy
Source: Nat Commun. 2024 Jun 26;15:5398. doi: 10.1038/s41467-024-49804-x (PMC11208445; doi:10.1038/s41467-024-49804-x)
Supplement: Supplementary file 3 — Description of Additional Supplementary Files [file 41467_2024_49804_MOESM3_ESM.docx]

Inventory of Supporting Information

**Supplementary Data 1.** Gene expression profiling revealed by heart RNA-sequencing analysis.

**Supplementary Data 2.** Summarized corresponding simplified molecular-input line-entry system (SMILES) representations of cationic compounds.

**Supplementary Data 3.** Gene expression profiling revealed by liver RNA-sequencing analysis.

**Supplementary Video S1.** The detailed description of workflow of the robotic-assisted microfluidics platform.
