# Supplementary material for: Comparative optimization of polysaccharide-based nanoformulations for cardiac RNAi therapy
Source: Nat Commun. 2024 Jun 26;15:5398. doi: 10.1038/s41467-024-49804-x (PMC11208445; doi:10.1038/s41467-024-49804-x)
Supplement: Supplementary file 8 — Reporting Summary [file 41467_2024_49804_MOESM8_ESM.pdf]

nature portfolio

Corresponding author(s): Hélder A. Santos

Last updated by author(s): 28th May, 2024

Reporting Summary

Nature Portfolio wishes to improve the reproducibility of the work that we publish. This form provides structure for consistency and transparency in reporting. For further information on Nature Portfolio policies, see our Editorial Policies and the Editorial Policy Checklist.

Statistics

For all statistical analyses, confirm that the following items are present in the figure legend, table legend, main text, or Methods section.

n/a	Confirmed	
☐	☒	The exact sample size (*n*) for each experimental group/condition, given as a discrete number and unit of measurement
☐	☒	A statement on whether measurements were taken from distinct samples or whether the same sample was measured repeatedly
☐	☒	The statistical test(s) used AND whether they are one- or two-sided<br>*Only common tests should be described solely by name; describe more complex techniques in the Methods section.*
☒	☐	A description of all covariates tested
☐	☒	A description of any assumptions or corrections, such as tests of normality and adjustment for multiple comparisons
☐	☒	A full description of the statistical parameters including central tendency (e.g. means) or other basic estimates (e.g. regression coefficient) AND variation (e.g. standard deviation) or associated estimates of uncertainty (e.g. confidence intervals)
☐	☒	For null hypothesis testing, the test statistic (e.g. *F*, *t*, *r*) with confidence intervals, effect sizes, degrees of freedom and *P* value noted<br>*Give P values as exact values whenever suitable.*
☒	☐	For Bayesian analysis, information on the choice of priors and Markov chain Monte Carlo settings
☒	☐	For hierarchical and complex designs, identification of the appropriate level for tests and full reporting of outcomes
☐	☒	Estimates of effect sizes (e.g. Cohen's *d*, Pearson's *r*), indicating how they were calculated

Our web collection on statistics for biologists contains articles on many of the points above.

Software and code

Policy information about availability of computer code

Data collection	BD LSR II Flow Cytometer, Zeiss LSM 710, PerkinElmer IVIS Spectrum, Bio-Rad ChemiDoc MP image system
Data analysis	Statistical analysis was performed on GraphPad Prism 9.4.1; Flow cytometry data was analyzed on FlowJo software (version 10.4); Western blotting was quantified by using ImageJ software; Confocal images were analyzed by Zeiss Zen 3.8.

For manuscripts utilizing custom algorithms or software that are central to the research but not yet described in published literature, software must be made available to editors and reviewers. We strongly encourage code deposition in a community repository (e.g. GitHub). See the Nature Portfolio guidelines for submitting code & software for further information.

Data

Policy information about availability of data

All manuscripts must include a data availability statement. This statement should provide the following information, where applicable:

- Accession codes, unique identifiers, or web links for publicly available datasets
- A description of any restrictions on data availability
- For clinical datasets or third party data, please ensure that the statement adheres to our policy

All data are available in the main text or the supplementary materials. Source data are provided with this paper.

Research involving human participants, their data, or biological material

Policy information about studies with human participants or human data. See also policy information about sex, gender (identity/presentation), and sexual orientation and race, ethnicity and racism.

Reporting on sex and gender	No human participants were involved in this study.
Reporting on race, ethnicity, or other socially relevant groupings	No human participants were involved in this study.
Population characteristics	No human participants were involved in this study.
Recruitment	No human participants were involved in this study.
Ethics oversight	No human participants were involved in this study.

Note that full information on the approval of the study protocol must also be provided in the manuscript.

Field-specific reporting

Please select the one below that is the best fit for your research. If you are not sure, read the appropriate sections before making your selection.

[x] Life sciences [ ] Behavioural & social sciences [ ] Ecological, evolutionary & environmental sciences

For a reference copy of the document with all sections, see nature.com/documents/nr-reporting-summary-flat.pdf

Life sciences study design

All studies must disclose on these points even when the disclosure is negative.

Sample size	No statistical methods were used to determine the sample size in the study. The sample sized are clearly described in each figure legend. The sample size were determined by allowable error size, accuracy, resources, and need for statistical analysis (generally n >=3 throughout all the studies).
Data exclusions	No animals and/or data were excluded.
Replication	All experiments were repeated for at least three times and experimental findings were reproducible.
Randomization	For in vivo study, animal groups were randomized by body weight. For other experiments, all samples were randomly allocated into experimental groups, as there was no covariate in the study design.
Blinding	All the investigators were blinded to group allocation during data collection and analysis.

Reporting for specific materials, systems and methods

We require information from authors about some types of materials, experimental systems and methods used in many studies. Here, indicate whether each material, system or method listed is relevant to your study. If you are not sure if a list item applies to your research, read the appropriate section before selecting a response.

Materials & experimental systems

n/a	Involved in the study	
[ ]	[x]	Antibodies
[ ]	[x]	Eukaryotic cell lines
[x]	[ ]	Palaeontology and archaeology
[ ]	[x]	Animals and other organisms
[x]	[ ]	Clinical data
[x]	[ ]	Dual use research of concern
[x]	[ ]	Plants

Methods

n/a	Involved in the study	
[x]	[ ]	ChIP-seq
[ ]	[x]	Flow cytometry
[x]	[ ]	MRI-based neuroimaging

Antibodies

Antibodies used	GAPDH monoclonal antibody (60004-1-Ig, Proteintech), HRP-conjugated Alpha Tubulin Monoclonal antibody (HRP-66031, Proteintech), IRF3 monoclonal antibody (66670-1-Ig, Proteintech), Phospho-IRF3 (Ser396) antibody (AF2436, Affinity Biosciences), Recombinant mouse Dectin-1 (ab217331, Abcam), Ly-6G (MAB1037, R&D Systems), DAPI (G1012, Servicebio), HRP-conjugated Affinipure Goat Anti-Rabbit IgG(H+L) (SA00001-2, Proteintech), HRP-conjugated Affinipure Goat Anti-Mouse IgG(H+L) (SA00001-1,

	Proteintech), PE/Cyanine7 anti-mouse CD45 (103113, Biolegend), Brilliant Violet 650TM anti- mouse/Human CD11b (101259, Biolegend), PerCP/Cyanine5.5 anti-mouse Ly-6G (127615, Biolegend), Brilliant Violet 421TM anti-mouse Ly-6C (128031, Biolegend), PE-CyTM7 Rat Anti-Mouse CD45R/B220 (552772, BD Pharmingen), BV786 Mouse Anti-Mouse NK-1.1 (740853, BD Pharmingen), PE anti-mouse CD3ε Antibody (100307, Biolegend), APC anti-mouse CD11c (117309, Biolegend), Zombie NIR (423105, Biolegend).
Validation	All antibodies were verified by the supplier and each lot has been quality tested. All the antibodies used are from commercial sources and have been validated by the vendors. Validation statements and data are available on the manufacturer's website.

Eukaryotic cell lines

Policy information about cell lines and Sex and Gender in Research

Cell line source(s)	The human THP-1 monocytic cell line (cat. TIB-202) and the murine RAW264.7 macrophage cell line (cat. TIB-71) were obtained from ATCC.
Authentication	A short tandem repeat DNA profiling method was used to authenticate the cell lines and the results were compared with reference database.
Mycoplasma contamination	All cell line were tested for mycoplasma contamination. No mycoplasma contamination was found.
Commonly misidentified lines (See ICLAC register)	No commonly misidentified cell lines were used.

Animals and other research organisms

Policy information about studies involving animals; ARRIVE guidelines recommended for reporting animal research, and Sex and Gender in Research

Laboratory animals	C57BL/6 mice (male, 8-10 weeks age, 20-25g) were purchased from The Jackson Laboratory maintained in 12 h light/dark cycle at a constant temperature (22 ± 2 °C), supplied with standard lab chow and water ad libitum.
Wild animals	No wild animal was used in this study.
Reporting on sex	Only male mice were used in this study.
Field-collected samples	The study did not involve samples collected from field.
Ethics oversight	All animal experiments were performed in compliance with the guidelines from the Institutional Animal Care and Use Committee (IACUC) of Hangzhou Medical College (Animal License No. SYXK (Zhe) 2019-0011, Approval No. ZUCLA-IACUC-20010180).

Note that full information on the approval of the study protocol must also be provided in the manuscript.

Plants

Seed stocks	No plants were involved in this study.
Novel plant genotypes	No plants were involved in this study.
Authentication	No plants were involved in this study.

Flow Cytometry

Plots

Confirm that:

- [x] The axis labels state the marker and fluorochrome used (e.g. CD4-FITC).
- [x] The axis scales are clearly visible. Include numbers along axes only for bottom left plot of group (a 'group' is an analysis of identical markers).
- [x] All plots are contour plots with outliers or pseudocolor plots.
- [x] A numerical value for number of cells or percentage (with statistics) is provided.

Methodology

Sample preparation	Cells were detached by scraper. Details of sample preparation are provided in Methods section. Briefly, tissues samples were collected and cut into small pieces, followed by digested by DMEM medium containing collagenase type IV, Dispase II and DNase (heart tissue), or DMEM medium containing collagenase type I, HAase and DNase (liver tissue). The above cell suspension was then filtered, centrifuged and lysed by lysis buffer. Single-cell suspensions were obtained and stained with antibodies according to the manufacturer's protocols, and analyzed by flow cytometry.
Instrument	BD LSR II
Software	FlowJo software (version 10.4)
Cell population abundance	The absolute cells >= 10000 were analyzed for fluorescent intensity in the defined gate.
Gating strategy	In general, for cellular uptake, cells were first gated on FSC/SSC to identify the Cy5 signal (See supplementary information). For tissue samples flow cytometry analysis, single cells were single cells were selected by FSC and SSC plots. Live cells were selected as defined by Live Dead Stain-negativity. Immune cells were gated by CD45+ cells. Monocytes were gated by CD45 +CD11b+Ly6G-Ly6c+ cells. Neutrophils were gated by CD45+CD11b+Ly6G+ cells. T cells were gated by CD45+CD11b-CD3+. B cells were gated by CD45+CD11b-B220+. NK cells were gated by CD45+CD11b-NK1.1+. Dendritic cells were gated by CD45 +CD11b-CD11c+MHC II+ cells. Detailed gating strategies were provided in the Supplementary Information Figure 11.

☒ Tick this box to confirm that a figure exemplifying the gating strategy is provided in the Supplementary Information.
